# Alpha-Linolenic Acid: An Omega-3 Fatty Acid with Neuroprotective Properties—Ready for Use in the Stroke Clinic?

**DOI:** 10.1155/2015/519830

**Published:** 2015-02-19

**Authors:** Nicolas Blondeau, Robert H. Lipsky, Miled Bourourou, Mark W. Duncan, Philip B. Gorelick, Ann M. Marini

**Affiliations:** ^1^Institut de Pharmacologie Moléculaire et Cellulaire, Université de Nice Sophia Antipolis, 660 route des Lucioles, Valbonne, 06560 Sophia Antipolis, France; ^2^CNRS, IPMC, 06560 Sophia Antipolis, France; ^3^Inova Neuroscience Institute, Inova Health System, Falls Church, VA 22042, USA; ^4^Division of Endocrinology, Diabetes & Metabolism, Department of Medicine, School of Medicine, University of Colorado Denver, Anschutz Medical Campus, Aurora, CO 80045, USA; ^5^Obesity Research Center, College of Medicine, King Saud University, Riyadh 11461, Saudi Arabia; ^6^Mercy Health Hauenstein Neurosciences & Department of Translational Science and Molecular Medicine, Michigan State University College of Human Medicine, Grand Rapids, MI, USA; ^7^Department of Neurology and Program in Neuroscience, Uniformed Services University of the Health Sciences, 4301 Jones Bridge Road, Bethesda, MD 20814, USA

## Abstract

Alpha-linolenic acid (ALA) is plant-based essential omega-3 polyunsaturated fatty acids that must be obtained through the diet. This could explain in part why the severe deficiency in omega-3 intake pointed by numerous epidemiologic studies may increase the brain's vulnerability representing an important risk factor in the development and/or deterioration of certain cardio- and neuropathologies. The roles of ALA in neurological disorders remain unclear, especially in stroke that is a leading cause of death. We and others have identified ALA as a potential nutraceutical to protect the brain from stroke, characterized by its pleiotropic effects in neuroprotection, vasodilation of brain arteries, and neuroplasticity. This review highlights how chronic administration of ALA protects against rodent models of hypoxic-ischemic injury and exerts an anti-depressant-like activity, effects that likely involve multiple mechanisms in brain, and may be applied in stroke prevention. One major effect may be through an increase in mature brain-derived neurotrophic factor (BDNF), a widely expressed protein in brain that plays critical roles in neuronal maintenance, and learning and memory. Understanding the precise roles of ALA in neurological disorders will provide the underpinnings for the development of new therapies for patients and families who could be devastated by these disorders.

## 1. Introduction

**Figure 1:**
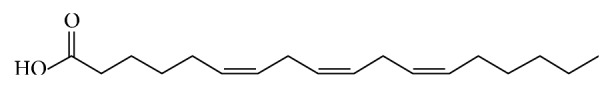
Structure of *α*-linolenic acid. *α*-Linolenic acid is an 18-carbon, polyunsaturated fatty acid that is essential for normal health. Because humans do not possess the enzymes to synthesize the compound, it must be obtained from dietary sources.

Dietary approaches for stroke prevention and rehabilitation hold promise to improve outcomes in individuals at risk of stroke and those who have had a stroke [1–4]. Although there is abundant literature that connects reduction in stroke risk to certain dietary elements and increase in stroke risk to other certain dietary components, there is a paucity of clinical trial data to direct the public and clinicians in this important area of clinical need. Compounds with pleiotropic effects aimed at reducing infarct size by one or more mechanisms and improving outcome would be advantageous in reducing the devastating effects of stroke on patients and their families [1–3]. One compound that has been demonstrated to exert neuroprotective, anti-inflammatory, and antidepressant properties is *α*-linolenic acid (ALA), an 18-carbon, essential omega-3 polyunsaturated fatty acid (PUFA) (Figure 1). In this review we discuss beneficial effects of *α*-linolenic acid and clinically relevant data to suggest that further exploration of this dietary component might be useful in stroke prevention and recovery.

Omega-3 fatty acids are required for normal health, especially for the brain development and function [16]. Prior work has shown that a seafood-rich diet was associated with low rates of coronary heart disease and autoimmune disorders in Greenland Eskimos which has been generally ascribed to the intake of eicosapentaenoic acid (EPA) and docosahexaenoic acid [DHA] [4]. A change in diet over the last century toward a higher total fat and saturated fat content and a sedentary lifestyle has been associated with an increase in the incidence of chronic disorders such as hypertension, diabetes, and atherosclerosis [17–20], all of which are stroke risk factors or risk markers. In addition, omega-6 fatty acids are consumed at a higher level compared with omega-3 fatty acids in a standard western diet and they have been implicated in coronary atherogenesis [21]. The distinction between the two types of PUFAs is underscored by the fact that a higher ratio of omega-6 fatty acids (linoleic acid) to omega-3 fatty acids (alpha-linolenic acid) increases platelet aggregation [22], is prothrombotic, and increases vasoconstriction [1–4, 20, 23]. These effects are presumably due, at least in part, to being integral components of the cell membrane [1, 24]. A large body of evidence from experimental, clinical, and epidemiologic research reports a cardioprotective role of long-chain omega-3 fatty acids EPA and DHA derived primarily from fatty fish. While prospective observational cohort investigations indicated that consumption of fatty fish twice or more a week significantly lowers risk of cardiovascular death [25], the findings from randomized clinical trials examining the effects of fish oil supplementation on cardiovascular disease morbidity and mortality in secondary prevention settings were inconsistent. Fourteen randomized clinical trials were evaluated by both Messori et al. [26] and Kwak et al. [27]. These two groups adopted different statistical methods, but neither found a benefit associated with omega-3 fatty acid supplements versus placebo [26, 27]. Importantly, however, the 14 randomized clinical trials so far reported have been small and short-term studies that were not specifically designed to evaluate CVD end points and, of note, the 2 large open-label trials that report a benefit with omega-3 supplementation [28, 29] were excluded from their analysis. While awaiting more definitive results that include a standardized dose and a formulation maximizing bioavailability, the American Heart Association has released dietary guidelines that recommend intake of fatty fish twice a week, underscoring the view that a cardioprotective diet needs to be rich in omega-3 fatty acids [30–32]. There is extensive literature on the effects of EPA and DHA in cardiovascular disease compared to *α*-linolenic acid, the precursor of EPA and DHA (see [1, 24] and the references therein).

## 2. Cardiovascular Disease (CVD) and ***α***-Linolenic Acid

In the absence of definitive evidence, several sources imply, rather than directly state, that the high ratio of omega-6/omega-3 that constitutes the typical western diet may promote the pathogenesis of many diseases, including cardiovascular disease, cancer, inflammatory and autoimmune diseases. It is therefore a widely held belief that restoring the balance omega-6/omega-3 to a ratio of 5 : 1 is important, but this “ratio theory” remains controversial. Indeed, a high omega-6 intake may not be characteristic of many western countries and a focus on the omega-6/omega-3 ratio risk diverts attention away from simply increasing the absolute intake of omega-3 fatty acids, which alone has been shown to have beneficial effects, especially on cardiovascular health [33]. Interestingly, only the daily intake of EPA and DHA was promoted while the absolute and relative change of omega-6/omega-3 in the food between the late paleolithic period and the current US western diet seems mainly mediated by the pronounced change in the linolenic acid (LA): *α*-linolenic acid (ALA) ratio of the diet [34]. This points out that the importance of ALA as a particularly bioactive component from vegetables food source has been underestimated, especially because humans, like all mammals, cannot synthesize *α*-linolenic acid (e.g., we do not possess the enzymes for* de novo* synthesis. ALA must therefore be obtained from the diet and excellent sources of ALA include rapeseed and walnuts [35, 36]. In fact, interest in omega-3 in CVD has mainly focused on EPA and DHA rather than ALA because ALA bioconversion to EPA and DHA is minimal and therefore a diet rich in ALA might not fulfill DHA requirements (for review, [37, 38]). Since a wide variety of protective mechanisms were ascribed directly to DHA (for review, [39, 40]), diet supplementation with high levels of ALA has been seen of little interest as compared to supplementation with preformed EPA or DHA. This might have been an unfortunate outcome in view of the growing evidence that dietary ALA may also protect against CVD.

First, ALA-enriched diets have been shown in some animal studies to influence the concentration of lipoprotein in plasma. This ability to decrease low density lipoprotein (LDL) may be of importance as increased levels of LDL in plasma are strikingly correlated with the risk of developing atherosclerosis and CHD. Unfortunately, this plasmatic LDL reduction has not been found in studies in humans, although consumption of ALA-enriched sources affected LDL content in ALA, EPA, and DHA that were increased [41–43]. Second, consumption of ALA-enriched sources and of fish oils rich in EPA/DHA has similar antiarrhythmic properties [44, 45], which are known to reduce the human risk of myocardial infarction and fatal ischemic heart disease. Nevertheless, the conclusion of prospective cohort studies that dietary ALA is beneficial against CVD [46–48] has been recently challenged by a meta-analysis concluding that increasing ALA intake may only produce modest cardioprotection [49]. In addition to the modification of ionic channels currents induced by the incorporation of these polyunsaturated fatty acids into the cardiomyocytes membrane phospholipid bilayer, which could account for the antiarrhythmic effects, omega-3 PUFAs are paradoxical antioxidant and anti-inflammatory compounds and therefore could indirectly decrease oxidation and inflammation associated with CVD [50–52]. A diet rich in ALA reduces proinflammatory cytokines which in turn is related to the omega-6/omega-3 ratio (i.e., a lower ratio reduces the proinflammatory mediators [7]; inflammation is considered to play an important role in atherosclerosis, a major risk factor for cardiovascular disease and stroke [53]). In a recent study, de Goede and colleagues [54] have examined the 10-year incidence of CHD and stroke in relation to ALA intake in a Dutch population-based cohort of over 20,000 adults. While no association between ALA intake and incident coronary heart disease was observed, their study revealed that ALA intake lowered the risk of stroke. Compared to an Eskimo population where the omega-6/omega-3 ratio is 1, the ratio of a typical western diet is 10/1–25/1 [34]. Thus, increasing the intake of ALA may be beneficial in reducing stroke risk.

## 3. Stroke and ***α***-Linolenic Acid

A typical western diet is severely deficient in omega-3 fatty acids and this may elevate the risk for stroke [1, 3, 24, 54]. During an ischemic stroke, glutamate excitotoxicity through overactivation of N-methyl-D-aspartate (NMDA) receptors is the major mechanism of neuronal cell death within the core and surrounding ischemic area called the penumbra. Neuronal necrosis driven by glutamate excitotoxicity occurs within minutes to hours following cerebral ischemia. This creates an extremely reduced time window of intervention for administration of therapeutics aimed at inhibiting glutamate-mediated cell death pathways [55]. This time constraint of acute neuroprotection will probably be difficult to achieve in clinical practice drawing attention to the importance of prevention. The common view of prevention of the risk factors is to reduce the occurrence of stroke. Nevertheless an emerging concept in the field is that nutritional factors may exert a protective role against stroke-induced damage, a field of study of potentially major relevance but still poorly addressed (see [1, 3]).

There is a great deal of evidence that ALA is a potent neuroprotective agent against focal and global ischemia in animal models [11, 56–62]. This same mechanism appears to underlie clinical findings, where, in adult men, serum levels of ALA were independently associated with a 37% reduction in stroke risk [63]. Also, the higher the intake of *α*-linolenic acid, the lower the prevalence of a carotid plaque [64], and similar results were reported in mice [35]. ALA activates a neuronal background rectifying potassium channel [65] leading to membrane hyperpolarization which in turn increases the magnesium block of the calcium channel associated with NMDA receptors which play a predominant role in mediating glutamate-mediated excitotoxic neuronal cell death [58, 61]. In this rodent model of global ischemia where hippocampal pyramidal neuronal death is mainly driven by glutamate excitotoxicity, we found that ALA exerted a profound protective effect that was more pronounced and reproducible than with EPA and DHA [61]. Additional studies in rodents revealed an essential role for the transcription factor, nuclear factor kappaB, in the ability of ALA to protect neurons against ischemia [11] and to induce tolerance [57], a phenomenon where neurons become resistant to a stressful environment such as ischemia [66]. ALA was shown to increase levels of brain-derived neurotrophic factor (BDNF), a widely distributed protein that [59] in the brain carries out diverse functions, including neuronal maintenance, learning and memory, neuronal survival, and neurogenesis [67–72]. Other proteins, such as HSP70, a heat shock protein [57, 60], which acts as a protein chaperone, also have roles in regulating programmed cell death (i.e., apoptosis) [73]. While some features are known, the precise mechanisms by which *α*-linolenic acid exerts its pleiotropic properties in brain are still not clear. Omega-3 fatty acids act via multiple mechanisms such as through the alteration of plasma membrane fluidity, lipid rafts, and signal transduction mechanisms in addition to effects on gene expression [74]. Delineating ALA-mediated mechanisms may increase the number of cellular and molecular targets that lead to enhanced therapeutic efficacy.

## 4. Stroke and Brain-Derived Neurotrophic Factor (BDNF)

**Figure 2:**
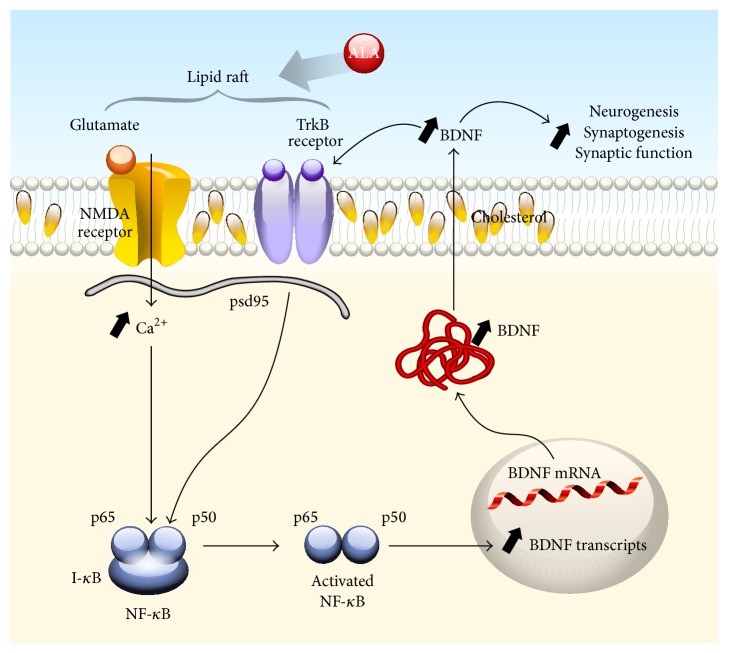
Interplay between NMDA and TrkB receptors mediated by ALA-induced lipid rafts in neuronal plasma membranes. An increase in the nutraceutical ALA is hypothesized to markedly increase membrane fluidity leading to the efficient formation of lipid rafts [5] in neuronal plasma membranes. Lipid rafts are the functional domains of the plasma membrane and play a crucial role in the regulation of transmembrane signaling [6]. TrkB receptors and some NMDA receptors are constituents of lipid rafts [7–10] and one of the major nonprotein components of lipid rafts is cholesterol [6]. The enhanced formation and/or efficiency of transmembrane signaling is hypothesized to result in enhanced activation (phosphorylation) of NMDA and TrkB receptors via the binding of BDNF to its cognate receptor, TrkB. Activation of NMDA receptors results in enhanced calcium influx and activation of signal transduction pathways leading to activation of nuclear factor kappa B (NF-*κ*B) via the canonical pathway (phosphorylation of I-*κ*B leads to its dissociation from the dimer (p65/p50) which then translocates to the nucleus where it binds to *κ*B sites to regulate gene expression) which in turn increases BDNF mRNA and protein levels [11–14]. Enhanced intracellular BDNF protein expression would lead to an increase in secretion, thereby maintaining its availability to bind to TrkB in an autocrine fashion [14, 15] as well as to stimulate neurogenesis, synaptogenesis, and synaptic function at distant sites (paracrine function).

Of the known gene targets of ALA, BDNF shows promise as a therapy for stroke. In many studies, BDNF has been shown to reduce infarct size and improve outcome (see [75–77] and the references therein) whereas blocking endogenous BDNF worsens ischemia [78]. Administration of BDNF via the intravenous route as well as the intracerebroventricular route reduced infarct size and improved outcome in the transient middle cerebral artery occlusion model of stroke [79, 80]. However, in humans, anticipated pharmacokinetic challenges make it difficult to develop BDNF itself as a therapy to the clinic [81]. This problem, however, creates opportunities to discover compounds that increase endogenous expression of BDNF in brain. To this end, chronic ALA treatment increases BDNF mRNA and protein levels in the cortex and hippocampus (Figure 2), two brain regions that are susceptible to ischemia but are also involved in plasticity responses. ALA increases neurogenesis, synaptogenesis, and synaptic function in the rodent brain [82]. The ability to increase neurogenesis in the brain is critical because it has been shown that neural stem cells improve neurological function in stroke [83–87]. Neural stem cells can modulate the ischemic environment via the upregulation of survival-promoting/neurotrophic factors such as BDNF and/or by restoring neurotransmitter function by integrating in existing networks and improving network circuitry. Taken together, these findings indicate that ALA induces tolerance and reduces infarct size in animal models of stroke. ALA was also demonstrated to exert antidepressant activity and increase BDNF mRNA and protein levels in the brain which in turn likely stimulates neurogenesis, synaptogenesis, and synaptic function. The benefit between the intake of ALA and the reduction in stroke risk in humans, the substantial evidence that ALA reduces infarct size, improves outcome and survival in animal models and the fact that ALA exhibits a wide safety margin provides a strong rationale for the systematic study of ALA administration in stroke.

## 5. Stroke, Depression, ALA, and BDNF

Poststroke depression is a common occurrence and can adversely affect outcome after stroke [88]. Stroke and depression are complex and multifaceted diseases but both disorders have common pathological substrates that could be targeted by therapeutic intervention. For example, there is growing evidence that neuroplasticity plays a crucial role in both pathologies. Consequently, compounds that increase neuroplasticity in the brain could ameliorate or prevent an infarct and reduce downstream consequences such as poststroke depression.

**Figure 3:**
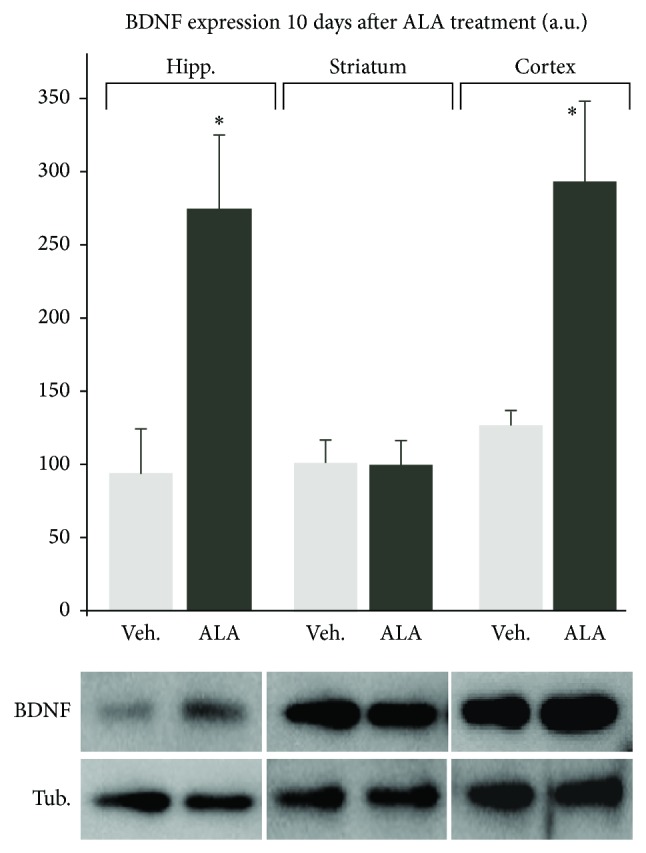
*In vivo* subchronic ALA treatment increases mature BDNF levels in neurons of the cortex and hippocampus, but not in striatum. BDNF increase in these specific brain regions is consistent with well-known properties for the efficiency of antidepressant drugs and with the level of brain protection offered by the subchronic ALA treatment. Mature BDNF expression was measured 10 days after the subchronic treatment by Western blots in cortex, hippocampus (^*^ *P* > 0.05), and striatum (*P* < 0.05) of mice injected with ALA or vehicle. Subchronic treatment consisted of three i.v. injections of 500 nmol/kg of *α*-linolenic acid on days 1, 3, and 7.

A longitudinal study of 50,000 women found that increased intake of ALA reduced depressive symptom [89]. Earlier studies showed similar results [90–92]. In normal mice, ALA treatment (given intravenously or in the diet) exerted an antidepressant effect. This effect was associated with increased synaptogenesis and an increase in BDNF mRNA levels in brain (Figure 3; [82, 93]). Evidence has shown that antidepressant drugs enhance the activation of TrkB receptors, the high affinity receptor that binds BDNF [94] and is a key event in exerting antidepressant properties [82, 94, 95]; BDNF has been implicated in mediating the antidepressant effects in brain [96].

## 6. Conclusion

In common with several others groups, we have demonstrated the broad neuroprotective and neuroplastic potential of omega-3 injection in animal models of neurodegenerative conditions, including acute neurological injuries such as stroke and spinal cord injury (for review, see [1, 3, 97]. In addition, intravenous perfusion of omega-3 fatty acid—in the form of 10% fish oil emulsion supplementing parenteral nutrition—has been shown to improve organ failure-related outcomes [98]. Although the impact of omega-3 fatty acid intravenous supplementation in human neurological conditions has not been addressed, it is tempting to speculate that this approach may offer significant benefit in human ischemic conditions. With regard to omega-3 consumption, a maximum dose of 3 g/day of long chain omega-3 fulfills the Generally Recognized as Safe status in the United States and the French recommendation not to exceed more than 15 times the Daily Recommended Intake [99]. Therefore, we believe that, in light of the currently available data, the conventional recommendations of omega-3 at a dose of 1 g/day of ALA, or 0.750–1 g/day of EPA + DHA, may offer therapeutic benefit in patients at risk of cardiovascular diseases. It is also noteworthy that these doses are without adverse effects. General consensus on the importance of eating for health may turn as a particular commitment for prevention, recovery, and rehabilitation from stroke. Healthy eating after stroke may be important for recovery though additional formal testing is needed, as it could be to improve outcome and reduce reoccurrence. Choosing healthy foods may be a challenge, underlying the importance of identifying natural products with health benefit, like ALA that is a nonproprietary, naturally occurring omega-3 fatty acid contained in foodstuffs. ALA has anti-inflammatory and other potential beneficial properties and, based on the weight of available data, may reduce stroke risk, size, and/or consequences. Sources of *α*-linolenic acid include but are not limited to flaxseed, rapeseed, and walnuts. ALA is well tolerated and can be supplemented into the diet in a variety of food sources including muffins. The potential benefits of ALA are supported by both animal studies and human observational epidemiologic studies. Early phase clinical trials evaluating *α*-linolenic acid are justified, and if these indicate benefit, larger scale studies of this agent in stroke prevention should follow.
